# Anticoagulant-Dependent Platelet Morphological Artefacts in Platelet-Rich Plasma Preparation: A Prospective Paired Study Comparing EDTA and Sodium Citrate with Implications for Orthobiologic Therapy

**DOI:** 10.3390/biomedicines14071578

**Published:** 2026-07-14

**Authors:** Luis García-Bordes, Lorenzo Escutia-Marí, Silvia Vizcaíno-Navarro, Patricia Laiz-Boada, Roberto Seijas-Vázquez, Pedro Álvarez-Díaz, Xavier Cuscó-Segarra, David Barastegui-Fernández, Miguel Vázquez-Gómez, Iker Ayestaran-Calero, Paula Velasco-Alcalde, Montserrat García-Balletbó, Miguel Azanarez-Jiménez, Ramón Cugat-Bertomeu

**Affiliations:** Instituto Cugat—Quirónsalud Barcelona, Plaza Alfonso Comín 4-6, 08036 Barcelona, Spain; lescutia@institutocugat.com (L.E.-M.); svizcaino@institutocugat.com (S.V.-N.); plaiz@institutocugat.com (P.L.-B.); rseijas@institutocugat.com (R.S.-V.); xcusco@institutocugat.com (X.C.-S.); dbarastegui@institutocugat.com (D.B.-F.); mvazquez@institutocugat.com (M.V.-G.); pvelasco@institutocugat.com (P.V.-A.); mgarcia@institutocugat.com (M.G.-B.); mazanarez@institutocugat.com (M.A.-J.); ramon.cugat@institutocugat.com (R.C.-B.)

**Keywords:** platelet-rich plasma, PRP, EDTA, sodium citrate, anticoagulant, mean platelet volume, platelet morphology, platelet distribution width, large platelet ratio, orthobiologics, regenerative medicine, orthopaedic surgery

## Abstract

**Background/Objectives:** The anticoagulant used for blood collection is a fundamental but underexplored variable in platelet-rich plasma (PRP) preparation. Ethylenediaminetetraacetic acid (EDTA) and sodium citrate act on platelets through distinct calcium chelation mechanisms with potentially different consequences for PRP quality. Our group has previously demonstrated that biological and demographic variables independently modulate platelet composition in PRP; the present study extends this analysis to the pre-analytical anticoagulant variable. No prospective paired clinical study has systematically compared the effects of EDTA and sodium citrate on platelet morphological parameters in a real clinical setting. This study aimed to characterise these differences and evaluate their implications for orthobiologic therapy. **Methods:** A prospective within-subject paired-sample study was conducted at Instituto Cugat—Quirónsalud Barcelona (November 2025–April 2026). Twenty-six consecutive adult patients undergoing routine blood extraction prior to orthopaedic procedures had blood drawn simultaneously into K_2_-EDTA and sodium citrate (3.2%) tubes. Full haematological analysis was performed on a Sysmex XN automated analyser within 30 min. Primary outcomes were mean platelet volume (MPV), platelet distribution width (PDW), large platelet ratio (P-LCR), large platelet cell count (P-LCC), and plateletcrit (PCT). Statistical comparisons used the paired *t*-test or Wilcoxon signed-rank test; effect sizes were quantified as Cohen’s d. **Results:** Seven of eight platelet-related parameters differed significantly between anticoagulants (all *p* < 0.001). Compared to sodium citrate, EDTA produced systematically higher MPV (+10.1%, d = 2.81), P-LCR (+25.8%, d = 2.41), P-LCC (+24.3%, d = 1.70), PDW (+13.5%, d = 1.33), PCT (+7.3%, d = 0.78), RDW-CV (+2.0%, d = 0.83), and RDW-SD (+2.6%, d = 0.80). MPV was higher with EDTA in all 26/26 paired samples without exception. Total platelet count did not differ significantly (*p* = 0.135). Effect sizes for all morphological parameters were large (d ≥ 0.78). **Conclusions:** EDTA is associated with large, reproducible, and universal platelet morphological changes consistent with calcium chelation-induced artefact, not genuine platelet hypertrophy. These artefactual changes systematically overestimate platelet size and large platelet indices by up to 26%, with direct implications for PRP quality assessment in orthobiologic medicine. Sodium citrate should remain the anticoagulant of choice for PRP preparation. Clinicians using EDTA must recognise that morphological parameters do not reflect functional platelet capacity.

## 1. Introduction

Platelet-rich plasma (PRP) is an autologous biological concentrate derived from whole blood by differential centrifugation, increasingly used across musculoskeletal medicine, orthopaedic surgery, and regenerative therapy. Its therapeutic rationale is the supraphysiological delivery of platelet-derived bioactive molecules—principally platelet-derived growth factor BB (PDGF-BB), transforming growth factor-beta 1 (TGF-β1), vascular endothelial growth factor (VEGF), and epidermal growth factor (EGF)—to target tissues, initiating anabolic and anti-inflammatory cascades that support tissue repair [1,2].

Despite substantial clinical adoption, PRP remains characterised by marked preparation heterogeneity, recognised as the principal obstacle to consistent clinical outcomes [3,4]. The sources of this variability are multiple and operate at different stages of the preparation pipeline: biological patient-level variables (age, sex, body mass index), procedural variables (centrifugation speed and time, activation method, leukocyte content), and pre-analytical variables (collection technique, tube type, anticoagulant). Our group has recently characterised the biological contribution to PRP variability in a single-centre retrospective cohort of patients with musculoskeletal pathologies, demonstrating that younger patients have higher platelet concentrations in both blood and PRP, that sex affects platelet concentration differently in blood versus PRP, and that intrasubject variability is significantly greater in PRP than in whole blood [5,6]. These findings confirm that even before any processing step, the compositional baseline of PRP is a moving target. The present study addresses the question of what the anticoagulant—the very first pre-analytical decision in PRP preparation—adds to this variability.

Anticoagulants prevent clotting by sequestering calcium, essential for the coagulation cascade. Sodium citrate (and ACD-A) are conventional choices for PRP, largely by tradition from platelet transfusion medicine [7,8]. Ethylenediaminetetraacetic acid (EDTA, K_2_ or K_3_ salts), the universal anticoagulant in clinical haematology, is generally excluded from PRP preparation workflows based on concerns about its irreversible, high-affinity calcium chelation and potential cytotoxicity [9]. However, the evidence base for this exclusion is fragile: existing comparative studies are predominantly in vitro, use non-clinical donor populations, and apply heterogeneous processing protocols that complicate interpretation [10,11]. Furthermore, studies published in this journal have reported contradictory findings: Takebe et al. found that EDTA caused platelet swelling and activation but yielded the highest platelet number [11], while Guo et al. subsequently demonstrated that despite apparent platelet activation markers, VEGF release after CaCl_2_ activation was substantially lower in EDTA-prepared PRP compared to citrate (265 vs. 629 pg/mL, *p* = 0.013) [12]. The functional paradox suggested by these in vitro data—more activation markers but less growth factor release—has never been evaluated in a clinical paired cohort. It is noted that this comparison lacks a no-anticoagulant control; it cannot be formally excluded that citrate actively facilitates VEGF production or release—for example, through interactions with membrane transporters or metabolic intermediates—rather than simply preserving a physiological baseline state.

EDTA tubes are universally available in every clinical setting at minimal cost, while dedicated citrate PRP kits can be expensive or unavailable in resource-limited environments. If EDTA produces morphologically altered but functionally comparable platelets, this would have significant practical implications for PRP accessibility. Conversely, if EDTA-induced morphological changes translate to functional impairment, clinicians using EDTA-prepared PRP may be unknowingly administering a suboptimal product—especially problematic given that automated haematological parameters from EDTA tubes are routinely used as PRP quality indicators.

The present study addresses this gap with a prospective, within-subject paired design at a high-volume orthopaedic centre. The primary objective was to systematically quantify differences in platelet morphological parameters between EDTA and sodium citrate anticoagulation in a real clinical cohort. Secondary objectives were to determine effect sizes, establish the consistency of the anticoagulant effect across individual subjects, and formulate evidence-based recommendations for anticoagulant selection in PRP-based orthobiologic therapy.

## 2. Materials and Methods

### 2.1. Study Design and Ethical Framework

A prospective, within-subject paired-sample observational study was designed and conducted at Instituto Cugat—Quirónsalud Barcelona between November 2025 and April 2026. The study was approved by the Institutional Review Board (Comité Ético de Investigación Grupo Hospitalario Quirónsalud-Catalunya) (reference: SET-PRP-2021-01) and conducted in full accordance with the Declaration of Helsinki (2013 revision). Written informed consent was obtained from all participants prior to any study procedure.

### 2.2. Participants

Consecutive adult patients scheduled for routine pre-operative or pre-procedural blood extraction prior to orthopaedic surgical or regenerative orthopaedic procedures were invited to participate. Inclusion criteria: (1) age ≥ 18 years; (2) no known haematological disorder; (3) no anticoagulant or antiplatelet therapy in the preceding 10 days; (4) no active systemic infection or fever; (5) no use of systemic corticosteroids in the preceding 4 weeks. Exclusion criteria: thrombocytopenia (PLT < 100 × 10^3^/µL), thrombocytosis (PLT > 600 × 10^3^/µL), known platelet function disorder, pregnancy, or any condition in which additional blood extraction was clinically contraindicated.

### 2.3. Blood Collection Protocol

For each enrolled participant, blood was obtained from a single antecubital venepuncture performed by a single trained phlebotomist using a 21-gauge needle with a vacuum collection system. Two tubes were filled in a fixed sequence: (1) K_2_-EDTA tube (1.8 mg/mL EDTA; blue cap, BD Vacutainer^®^ (Becton Dickinson, Franklin Lakes, NJ, USA)) and (2) sodium citrate tube (3.2% trisodium citrate, blood-to-anticoagulant ratio 9:1; blue cap, BD Vacutainer^®^). EDTA was always collected first to avoid anticoagulant carryover effects and to minimise venous stasis effects on platelet activation. This sequence follows the standard order of draw recommended by the Clinical and Laboratory Standards Institute (CLSI H3-A6, 2007) [13], which places citrate tubes after EDTA tubes to prevent calcium contamination of the citrate sample. The interval between EDTA and citrate tube filling was less than 60 s in all cases, a timeframe insufficient to produce meaningful platelet activation changes that could confound morphological measurements. Immediately after collection, both tubes were inverted gently 8–10 times to ensure homogeneous mixing. All samples were transported at room temperature and processed without delay.

### 2.4. Haematological Analysis

Both tubes from each participant were analysed on the same calibrated Sysmex XN-series automated haematology analyser (Sysmex Corporation, Kobe, Japan) within 30 min of collection. The analyser underwent daily internal calibration and external quality control according to institutional protocols and ISO 15189 (Sysmex Corporation, Kobe, Japan) accreditation requirements. Parameters recorded: white blood cell count (WBC) with five-part differential; red blood cell count (RBC); haemoglobin (HGB); haematocrit (HCT); mean corpuscular volume (MCV); mean corpuscular haemoglobin (MCH, MCHC); red cell distribution width by coefficient of variation (RDW-CV) and standard deviation (RDW-SD); platelet count (PLT); mean platelet volume (MPV); platelet distribution width (PDW); plateletcrit (PCT); large platelet ratio (P-LCR); and large platelet cell count (P-LCC).

### 2.5. Statistical Analysis

All statistical analyses were performed using IBM SPSS Statistics v26.0 (IBM Corp., Armonk, NY, USA) and Python 3.11 with SciPy 1.11. Sample size was determined prospectively based on mean platelet volume (MPV) as the primary outcome. Drawing on published data for MPV differences between anticoagulants [9,14], a minimum clinically relevant mean difference in δ = 0.5 fL was assumed, with a conservative standard deviation of paired differences of 0.9 fL. A two-tailed paired *t*-test with α = 0.05 and power of 80% yielded a required sample size of n = 26 pairs (n = (Zα/2 + Zβ)^2^ × SD^2^/δ^2^ = (1.96 + 0.84)^2^ × 0.81/0.25 ≈ 26). The enrolled sample of N = 26 pairs exactly meets this a priori requirement for the primary outcome.

For each paired variable, normality of within-pair differences was assessed using the Shapiro–Wilk test. Normally distributed differences (Shapiro–Wilk *p* > 0.05) were compared using the paired Student’s *t*-test; non-normally distributed variables using the Wilcoxon signed-rank test. Results are presented as mean ± standard deviation with mean difference and 95% confidence interval. Pearson correlation was used to evaluate the relationship between PLT and MPV within each anticoagulant group. Effect sizes were calculated as Cohen’s d from paired differences and interpreted as small (<0.5), medium (0.5–0.8), or large (>0.8).

The significance threshold was set at α = 0.05 (two-tailed). No correction for multiple comparisons was applied, for the following explicitly stated reasons. First, the platelet morphological indices examined (MPV, PDW, P-LCR, P-LCC, PCT) are mathematically dependent rather than statistically independent: PCT is the arithmetic product of PLT and MPV (PCT = PLT × MPV/10,000); P-LCR and P-LCC are derived from the same large-platelet size threshold as MPV; and PDW is a statistical dispersion measure of the same platelet size distribution from which MPV is derived. Applying a Bonferroni correction under the assumption of independence would therefore be statistically inappropriate. Second, these indices constitute a pre-specified, a priori family of measures representing a single biological construct (platelet size and morphology), not a set of independent hypotheses. As a sensitivity analysis, we verified that a Bonferroni-corrected threshold of α = 0.05/8 = 0.00625 would not alter any conclusion, since all significant *p*-values are <0.001.

## 3. Results

### 3.1. Participant Characteristics

Twenty-six participants were enrolled and completed the study (N = 26 paired samples; 13 male, 13 female). No participant was excluded after enrolment, and no sample was invalid or haemolysed. Clinical indications for blood extraction included: pre-operative assessment prior to knee arthroscopy or osteotomy (n = 14, 53.8%), pre-operative assessment for hip arthroscopy (n = 7, 26.9%), and regenerative PRP therapy for musculoskeletal conditions (n = 5, 19.2%). All samples were processed within the 30 min protocol window.

### 3.2. Platelet Count: Equivalent Between Anticoagulants

Total platelet count did not differ significantly between EDTA and citrate tubes (EDTA: 246.6 ± 56.6 × 10^3^/µL; citrate: 253.4 ± 67.3 × 10^3^/µL; mean difference −6.8 × 10^3^/µL, 95% CI −16.6 to 3.0; Wilcoxon *p* = 0.135; Cohen’s d = −0.28). This finding demonstrates that the two anticoagulants produce equivalent platelet enumeration in this clinical cohort, and that any differences in morphological parameters cannot be attributed to differences in the number of platelets analysed.

### 3.3. Platelet Morphological Parameters: Systematic, Large, and Universal Differences

In contrast to platelet count, all platelet morphological indices were significantly higher in EDTA-anticoagulated samples compared to sodium citrate (*p* < 0.001 for all seven parameters; Table 1). Critically, the direction of the effect was without exception: EDTA produced higher MPV in all 26 of 26 paired samples, confirming that the anticoagulant effect on platelet morphology is universal and not subject to individual biological variation.

**Table 1:** Paired comparison of haematological parameters between EDTA and sodium citrate anticoagulation (N = 26).

Parameter	Unit	EDTA Mean ± SD	Citrate Mean ± SD	Mean Difference (95% CI)	*p*-Value	Cohen’s d
Platelet count (PLT)	×10^3^/µL	246.6 ± 56.6	253.4 ± 67.3	−6.8 (−16.6 to 3.0)	0.135 (NS)	−0.28
Mean platelet volume (MPV)	fL	9.21 ± 1.14	8.37 ± 1.08	+0.84 (0.72 to 0.96)	<0.001 ***	2.81
Platelet distribution width (PDW)	fL	11.07 ± 2.05	9.75 ± 2.35	+1.32 (0.92 to 1.72)	<0.001 ***	1.33
Plateletcrit (PCT)	%	0.224 ± 0.045	0.208 ± 0.049	+0.015 (0.007–0.023)	<0.001 ***	0.78
Large platelet ratio (P-LCR)	%	22.04 ± 7.93	17.52 ± 7.11	+4.53 (3.77 to 5.29)	<0.001 ***	2.41
Large platelet cell count (P-LCC)	×10^9^/L	52.0 ± 13.9	41.8 ± 11.8	+10.2 (7.75 to 12.6)	<0.001 ***	1.70
RDW—CV (RDW-CV)	%	12.62 ± 0.62	12.37 ± 0.54	+0.25 (0.13 to 0.37)	<0.001 ***	0.83
RDW—SD (RDW-SD)	fL	47.20 ± 3.08	45.99 ± 2.71	+1.21 (0.60 to 1.82)	<0.001 ***	0.80

**Figure 1:**
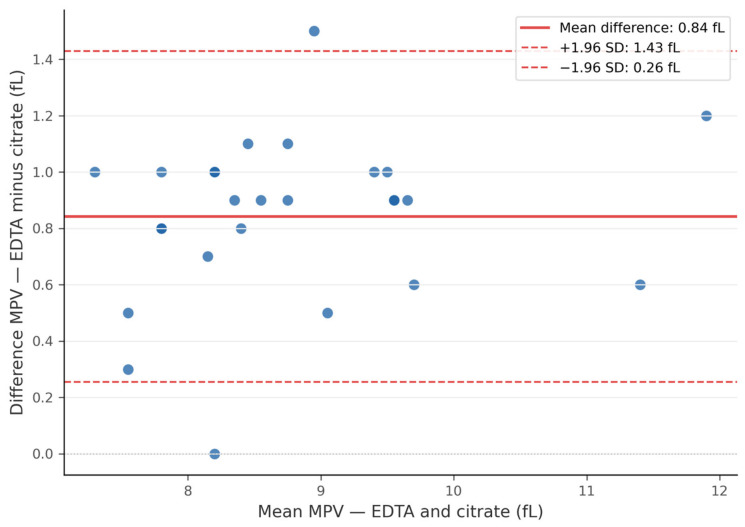
Bland–Altman plot for mean platelet volume (MPV): agreement EDTA (blue) anticoagulation (N = 26 paired samples). The solid red line represents the mean difference (+0.84 fL); dashed lines indicate the 95% limits of agreement. All 26 data points lie above zero, confirming systematic positive bias of EDTA over citrate across the full range of platelet sizes.

**Figure 2:**
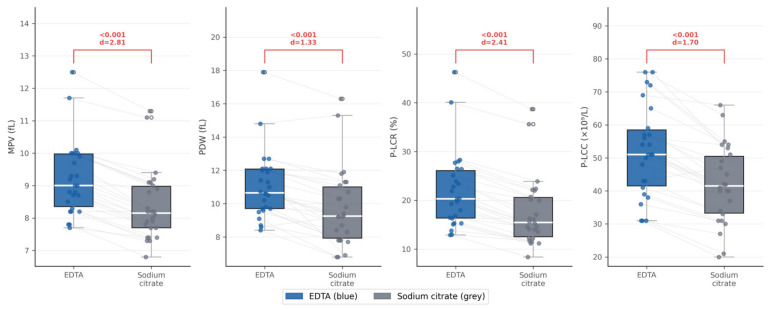
Box plots with individual paired data points for the four primary platelet morphological parameters: MPV, PDW, P-LCR, and P-LCC. Blue: EDTA; grey: sodium citrate. Grey lines connect values from the same participant. All *p* < 0.001 (Wilcoxon signed-rank test). Cohen’s d is shown above each panel.

**Figure 3:**
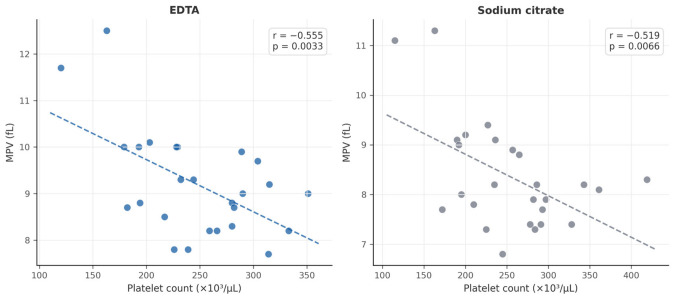
Pearson correlation between platelet count (PLT) and mean platelet volume (MPV) by anticoagulant (N = 26 pairs). Blue dots and dashed line: EDTA (r = −0.555, *p* = 0.003); grey dots and dashed line: sodium citrate (r = −0.519, *p* = 0.007); Dashed lines represent linear regression fits. The inverse PLT–MPV relationship is preserved under both anticoagulants, but the MPV distribution is systematically shifted upward by ~0.84 fL with EDTA.

**Figure 4:**
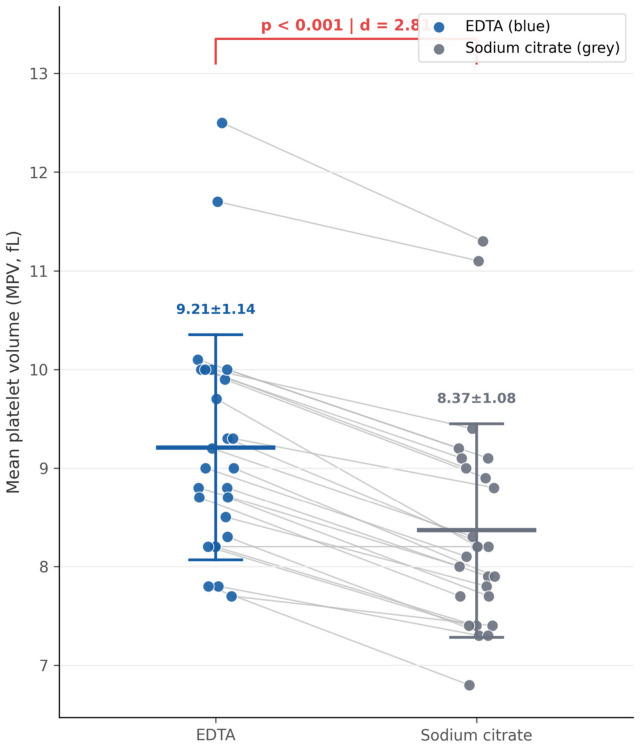
Paired dot plot showing individual MPV values for all 26 participants. Each grey line connects paired measurements from the same patient. Blue dots: EDTA; grey dots: sodium citrate. EDTA produced higher MPV in all 26/26 pairs without exception (*p* < 0.001; d = 2.81). Horizontal bars indicate group means ± SD.

Data presented as mean ± standard deviation; 95%CI: confidence interval of mean difference (EDTA minus citrate). Statistical test: paired Student’s *t*-test (Shapiro–Wilk *p* > 0.05) or Wilcoxon signed-rank test (*p* ≤ 0.05). Cohen’s d was calculated from paired differences. *** *p* < 0.001. NS: not significant (*p* = 0.135). Figure 1: Bland–Altman plot MPV. Figure 2: Box plots MPV, PDW, P-LCR, P-LCC. Figure 3: PLT vs. MPV correlation. Figure 4: Paired dot plot MPV, all 26 subjects.

Mean platelet volume (MPV) was 10.1% higher with EDTA (9.21 ± 1.14 fL vs. 8.37 ± 1.08 fL; mean difference +0.84 fL, 95%CI 0.72 to 0.96; *p* < 0.001; d = 2.81). A Cohen’s d of 2.81 is exceptional in haematological research, confirming that the EDTA effect on MPV is robust, reproducible, and far larger than what could be attributed to measurement variability. Bland–Altman analysis confirmed systematic positive bias of EDTA over citrate for MPV across the full range of platelet sizes, with limits of agreement of +0.84 ± 1.46 fL (Figure 1).

Platelet distribution width (PDW) was 13.5% higher with EDTA (11.07 ± 2.05 fL vs. 9.75 ± 2.35 fL; mean difference +1.32 fL, 95%CI 0.92 to 1.72; *p* < 0.001; d = 1.33), indicating that EDTA-induced swelling is not uniform across the platelet population but generates greater size heterogeneity. The large platelet ratio (P-LCR) was 25.8% higher with EDTA (22.04 ± 7.93% vs. 17.52 ± 7.11%; mean difference +4.53%, 95%CI 3.77 to 5.29; *p* < 0.001; d = 2.41), and the absolute large platelet count (P-LCC) was 24.3% higher (52.0 ± 13.9 vs. 41.8 ± 11.8 ×10^9^/L; mean difference +10.2 × 10^9^/L, 95%CI 7.75 to 12.6; *p* < 0.001; d = 1.70).

Plateletcrit (PCT) was 7.3% higher with EDTA (0.224 ± 0.045% vs. 0.208 ± 0.049%; *p* < 0.001; d = 0.78), consistent with higher MPV at equivalent PLT count. Red cell distribution width indices (RDW-CV: +2.0%, d = 0.83; RDW-SD: +2.6%, d = 0.80; both *p* < 0.001) were also elevated with EDTA, confirming a generalised morphometric effect extending beyond the platelet lineage.

The inverse correlation between PLT count and MPV was present under both anticoagulants (EDTA: r = −0.555, *p* = 0.003; citrate: r = −0.519, *p* = 0.007), indicating that the underlying biological relationship between platelet count and size is preserved—but the entire MPV distribution is systematically upward-shifted by approximately 0.84 fL with EDTA (Figure 3). Box plots with individual paired data for the four primary parameters are presented in Figure 2.

Full descriptive statistics, confidence intervals, test statistics, *p*-values, and effect sizes for all parameters are presented in Table 1.

## 4. Discussion

This prospective within-subject paired study demonstrates, for the first time in a real clinical orthopaedic cohort, that EDTA anticoagulation produces large, consistent, and universal elevations in all platelet morphological indices—MPV, PDW, P-LCR, P-LCC, PCT, RDW-CV, and RDW-SD—without a corresponding increase in total platelet count. The effect sizes (Cohen’s d = 2.81 for MPV, d = 2.41 for P-LCR) are exceptional by any standard in haematological research. Most strikingly, the EDTA effect on MPV was present in all 26 of 26 paired samples without exception, establishing that this is a universal biological response to EDTA chelation and not an individual-level phenomenon. This finding extends and complements our group’s previous characterisation of intrasubject and intersubject variability in PRP composition at the biological level [5], now identifying the anticoagulant as an additional pre-analytical source of systematic morphological bias that operates independently of patient biology.

### 4.1. Mechanism: Calcium Chelation Kinetics as the Driver of Platelet Swelling

The biological mechanism underlying these findings lies in the differential calcium chelation properties of the two anticoagulants. Sodium citrate forms competitive, reversible complexes with Ca^2+^ (thermodynamic stability constant *K*_a_ ~ 10^6^), maintaining residual free calcium and allowing platelets to remain in a physiological resting state. EDTA chelates calcium with far greater avidity and thermodynamically much stronger binding (*K*_a_ ~ 10^13^), with a very slow Ca^2+^ dissociation rate under physiological conditions, depleting both extracellular and partially intracellular free Ca^2+^ reserves [15]. Calcium is the central second messenger of platelet physiology: cytoskeletal organisation, shape change, pseudopod retraction, and granule secretion are all Ca^2+^-dependent processes governed by calmodulin, calpain, and the integrin αIIbβ3 (GP IIb/IIIa) complex [14]. When EDTA abruptly depletes the calcium pool, platelets undergo a conformational cascade—cytoskeletal rearrangement, osmotic rehydration, and membrane phosphatidylserine exposure—resulting in morphologically enlarged, heterogeneous platelets [16]. This is precisely the pattern reflected in the elevated MPV, PDW, and P-LCR values we report. It should be noted that EDTA chelates other divalent cations in addition to Ca^2+^, including Mg^2+^ and Mn^2+^, with similarly high affinities. While our data do not allow formal exclusion of a contribution from Mg^2+^ or Mn^2+^ depletion to the observed morphological changes, Ca^2+^ is the best-supported primary mediator given its dominant role as second messenger in platelet shape change and activation signalling. Confirmation of the relative contributions of individual cation depletion would require selective chelation experiments beyond the scope of the present study.

### 4.2. The Functional Paradox: Morphological Artefact Versus Biological Superiority

Mean platelet volume is widely endorsed as a surrogate of platelet metabolic activity and growth factor content: larger physiological platelets contain more α-granules and produce greater quantities of PDGF-BB, TGF-β1, VEGF, and EGF [17,18]. This relationship has been proposed as a basis for selecting processing conditions that maximise PRP potency. Our data show that EDTA produces platelets with a 10% higher MPV and a 26% higher P-LCR—values that, if accepted uncritically, would suggest a substantially more potent PRP.

The biological significance of these morphological parameters for PRP therapeutic activity rests on a well-established relationship: physiologically larger platelets contain more alpha-granules and dense granules, produce greater quantities of PDGF-BB, TGF-β1, VEGF, and EGF, and are considered more metabolically active [16,17]. MPV, PDW, and P-LCR are therefore widely used as surrogate quality indicators of PRP growth factor potential. The central finding of the present study is that EDTA artificially elevates these indicators through calcium chelation-induced platelet swelling, thereby decoupling morphological appearance from functional capacity. A clinician using EDTA-derived MPV or P-LCR to validate PRP quality will observe apparently superior morphological indices, while the preparation may have impaired degranulation capacity. This mechanistic chain—anticoagulant chelation → platelet swelling → artefactually elevated morphological indices → compromised Ca^2+^-dependent degranulation → reduced growth factor release—is directly supported by the functional evidence of Guo et al. [12], who demonstrated that EDTA-prepared PRP releases less than half the VEGF of citrate-prepared PRP after CaCl_2_ activation (265 vs. 629 pg/mL, *p* = 0.013), despite exhibiting higher surface activation markers.

However, this enlargement is artefactual, and the available functional evidence directly contradicts any interpretation of superiority. Guo et al. [12], in the most methodologically complete anticoagulant comparison study published in this journal to date, demonstrated that despite higher platelet counts and greater surface expression of activation markers (CD62P and PAC-1), EDTA-prepared PRP produced a VEGF concentration after CaCl_2_ activation that was less than half that of citrate-prepared PRP (265 vs. 629 pg/mL, *p* = 0.013). Takebe et al. [11] similarly confirmed that EDTA causes platelet swelling and activation in pure PRP. The mechanistic explanation is straightforward: EDTA’s thermodynamically strong Ca^2+^ chelation (slow dissociation kinetics) disrupts the Ca^2+^-dependent signalling cascades required for α-granule fusion with the plasma membrane and growth factor exocytosis, and this impairment is not fully reversible by exogenous CaCl_2_ addition at activation [19].

This creates a clinically dangerous paradox: an automated haematology report from an EDTA tube shows elevated MPV, PDW, and P-LCR—parameters increasingly used to validate PRP quality—while the functional platelet capacity for growth factor release may be substantially reduced. Our study quantifies the magnitude of this morphological artefact for the first time in a clinical paired cohort: up to 26% overestimation of the large platelet fraction, with effect sizes (d > 2.4) that far exceed what could be attributed to instrument variability.

A further dimension of this paradox emerges when our findings are considered alongside complementary work by our group published in The Archives of Bone and Joint Surgery [6]. That study, conducted using sodium citrate anticoagulation and the Endoret PRGF system in 877 patients, demonstrated that centrifugation at 580 g significantly reduces MPV compared to whole blood (9.8 fL in blood vs. 7.4 fL in PRP; *p* < 0.001), with low intrasubject variability [6]. This reduction reflects a biological selection process: centrifugation preferentially retains smaller, structurally intact, and functionally competent platelets in the PRP fraction. EDTA acts in precisely the opposite direction—it artificially enlarges platelet morphology without improving functional capacity. These two effects are mechanistically antithetical, yet both act on the same parameter (MPV) in ways that are invisible to the clinician who receives only an automated numerical report.

It is important to consider whether the elevated EDTA values or the lower citrate values better reflect the true physiological platelet state. Several converging but circumstantial observations are consistent with EDTA as the more likely source of the difference, while formally acknowledging that proof would require a no-anticoagulant reference control. First, the established normal MPV range in healthy adults is 7.2–11.7 fL [20]; our citrate values (mean 8.37 fL) fall centrally within this range, while EDTA values (mean 9.21 fL) are systematically above the midpoint. Second, our companion study in 877 patients using citrate anticoagulation demonstrated MPV of 9.87 fL in whole blood and 7.43 fL in PRP after centrifugation—both physiologically plausible [6]. Third, the consistent elevation of RDW in erythrocytes—cells that play no role in PRP—under EDTA but not citrate confirms a generalised multi-cellular morphometric effect of EDTA chelation, not a specific platelet response. Fourth, functional evidence from Guo et al. [12] shows that EDTA-prepared PRP yields less than half the VEGF of citrate-prepared PRP after activation, inconsistent with a scenario in which citrate suppresses platelet function.

The clinical consequence of this artefact is underappreciated. A clinician who measures MPV from an EDTA tube as a baseline quality indicator and then prepares PRP using citrate will observe a spuriously large apparent reduction in MPV between measurements—not because centrifugation has damaged the platelets, but because the EDTA baseline was artefactually inflated. This misinterpretation is directly relevant to PRP classification systems that encode platelet morphological parameters as quality metrics. An EDTA-derived MPV baseline used as input to such systems will systematically misclassify PRP quality, potentially leading to incorrect protocol adjustments or erroneous conclusions about centrifugation efficacy.

### 4.3. Platelet Count Equivalence: Reassuring but Insufficient

The absence of a significant PLT count difference (*p* = 0.135) contrasts with some prior literature reporting higher PLT count with EDTA [10,11], likely attributable to our strict 30 min processing window, minimising time-dependent aggregation effects. This is reassuring in that it confirms EDTA does not produce a clinically meaningful difference in available platelet number in a well-controlled protocol. However, PLT equivalence in no way implies therapeutic equivalence. Our group has previously shown [5] that PLT concentration in PRP exhibits high intrasubject variability (CV 27.85% in PRP vs. 21.32% in blood, *p* < 0.001) and that the platelet concentration ratio between blood and PRP is modulated by sex and BMI. Taken together, these data suggest that PLT is an insufficient and potentially misleading sole quality indicator for PRP—and that morphological parameters, particularly MPV and P-LCR, carry critical additional information that must be interpreted with anticoagulant context in mind.

### 4.4. RDW Elevation: Evidence of a Generalised Cellular Effect

The statistically significant elevation of RDW-CV (+2.0%) and RDW-SD (+2.6%) with EDTA provides important mechanistic corroboration. Erythrocytes are removed during PRP preparation and have no therapeutic role, yet their morphological indices are also systematically altered by EDTA through comparable osmotic mechanisms [21]. The observation that both platelets and erythrocytes show consistent EDTA-induced morphometric changes—while citrate affects neither—strongly supports the hypothesis that the platelet morphological changes we report are a direct consequence of EDTA’s calcium chelation properties, not a biological signal of platelet activation or superior quality. It is acknowledged, however, that erythrocytes and platelets are biologically distinct cell types, and this observation cannot formally establish mechanistic equivalence between EDTA effects on erythrocytes and platelets.

### 4.5. Clinical Recommendations for Anticoagulant Selection

Based on the totality of evidence—the present paired clinical study, the functional in vitro data of Guo et al. [12] and Takebe et al. [11], and the mechanistic literature on calcium chelation—sodium citrate (or ACD-A where available) should remain the anticoagulant of choice for PRP preparation in all settings where platelet functional integrity is the therapeutic objective. This recommendation is supported by: (1) the absence of artefactual morphological changes with citrate; (2) superior VEGF release capacity in citrate-prepared PRP; and (3) the universal, exception-free nature of EDTA-induced morphological distortion demonstrated across all 26 subjects of the present study.

EDTA may represent an acceptable pragmatic alternative in resource-limited settings, provided that three conditions are met: (1) blood processing is initiated within 30 min of collection; (2) PRP is activated with exogenous CaCl_2_ (10%, 50 µL per mL of PRP) immediately prior to injection to partially restore the calcium pool; and (3) clinicians explicitly discount automated morphological parameters—MPV, PDW, P-LCR, P-LCC, PCT—when using EDTA samples for PRP quality assessment, recognising that these values are artefactually elevated by 10–26% relative to the physiological state.

### 4.6. A Protocol Framework for the Scientific Community

The collective evidence from the present study, the functional in vitro data of Guo et al. [12] and Takebe et al. [11], and the morphological characterisation of our group [5,6] converges on a set of actionable recommendations that we propose as a minimum standard framework for anticoagulant selection, PRP quality reporting, and longitudinal monitoring in regenerative orthobiologic medicine.

Recommendation 1—Mandatory anticoagulant: sodium citrate (3.2% or 3.8%) or ACD-A must be the designated anticoagulant for all PRP preparations intended for clinical or research use. This is not merely a conventional preference: it is now supported by paired clinical evidence demonstrating that EDTA induces large, universal, and artefactual changes in platelet morphological parameters (Cohen’s d up to 2.81) that render automated haematological reports unreliable as quality indicators.

Recommendation 2—Mandatory flagging of EDTA-derived data: if EDTA is used for any reason—including parallel haematological analysis or resource-limited settings—all platelet morphological parameters derived from EDTA samples (MPV, PDW, P-LCR, P-LCC, PCT) must be explicitly flagged as anticoagulant-dependent artefacts and excluded from PRP quality classification. The only EDTA-derived parameter that retains validity for PRP purposes is total platelet count (PLT), which we confirm is equivalent between anticoagulants (*p* = 0.135).

Recommendation 3—Mandatory compositional reporting: PRP quality reporting in clinical research must specify: (a) the anticoagulant used; (b) the time elapsed between blood collection and analysis; (c) the analyser platform; and (d) which morphological parameters were used for quality classification and under which anticoagulant conditions. Without this information, cross-study comparisons of PRP quality are methodologically invalid. This recommendation is consistent with existing calls for PRP standardisation in the literature [3,4] but adds the anticoagulant as a mandatory reporting item that has hitherto been systematically omitted.

Recommendation 4—Citrate-based longitudinal quality monitoring: intrasubject reproducibility of MPV under a standardised citrate protocol is high—our group demonstrated a coefficient of variation in only 3.87% across multiple PRP doses in 298 patients [6]. This supports the use of MPV as a reliable longitudinal quality marker in clinical PRP programmes, provided citrate anticoagulation is consistently employed. The same cannot be assumed for EDTA, where morphological artefacts introduce systematic bias of a magnitude (10–26%) that substantially exceeds this biological intrasubject variability.

Adoption of these four recommendations—anticoagulant standardisation, explicit flagging of EDTA-derived morphological data, mandatory compositional reporting, and citrate-based longitudinal monitoring—would substantially reduce the methodological heterogeneity that continues to undermine the comparability and reproducibility of PRP research worldwide. We call on journal editors, PRP kit manufacturers, and clinical guideline bodies to incorporate these minimum standards into future PRP research and practice guidelines.

### 4.7. Strengths, Limitations and Future Directions

The primary methodological strength is the within-subject paired design, which eliminates the dominant source of noise in anticoagulant comparison studies—interindividual biological variability. By obtaining EDTA and citrate samples simultaneously from the same venepuncture, any observed difference is unambiguously attributable to the anticoagulant. The N = 26 pairs yielded extraordinary statistical power given the large effect sizes (post hoc power > 99.9% for all significant parameters). The finding of 26/26 directional consistency for MPV is itself a compelling statistical argument requiring no formal test. A single validated analyser was used throughout, eliminating inter-instrument variability. All patients were from a real orthopaedic clinical setting, enhancing external validity for the target clinical audience.

The principal limitation is the absence of functional platelet assays. While MPV, PDW, and P-LCR are validated surrogates of platelet biology, direct measurement of PDGF-BB, TGF-β1, and VEGF by ELISA following CaCl_2_ activation of paired EDTA- versus citrate-prepared PRP would be required to definitively translate morphological findings into therapeutic guidance. Flow cytometric assessment of CD62P expression and PAC-1 binding at baseline and after activation would further clarify the functional state of EDTA-affected platelets. A follow-up study incorporating these endpoints is planned at our centre, and will include: (a) ELISA-based quantification of PDGF-BB, TGF-β1, and VEGF in the supernatant of paired EDTA- and citrate-prepared PRP after standardised CaCl_2_ activation; and (b) flow cytometric assessment of CD62P expression and PAC-1 binding as markers of platelet activation state at baseline and after activation. We propose that the present morphological characterisation and the planned functional study constitute a two-phase research programme, with the current paper providing the essential quantitative foundation for the design and power calculation of the functional follow-up. The single-centre design and absence of demographic data stratification are additional limitations. Critically, the absence of a no-anticoagulant or immediate-fixation reference standard means that the formal directionality of the observed difference—whether EDTA inflates, citrate suppresses, or both contribute—cannot be definitively established from the present data alone. This constitutes a fundamental limitation of any two-anticoagulant comparison design and is acknowledged accordingly. The fixed EDTA-first tube collection order, while consistent with the CLSI H3-A6 standard and justified by the need to prevent calcium carryover into the citrate tube, introduces a potential draw-order effect that cannot be formally excluded. Future studies should consider a randomised crossover design alternating tube order between participants (with a discard tube for citrate-first participants) to eliminate this potential confound.

## 5. Conclusions

This prospective paired clinical study establishes, for the first time in a real orthopaedic cohort, that EDTA anticoagulation induces large, systematic, and universal artefactual elevations in platelet morphological parameters—including a 10% increase in mean platelet volume (d = 2.81), a 26% increase in the large platelet fraction (d = 2.41), and a 14% increase in platelet size heterogeneity (d = 1.33)—compared to sodium citrate, in all 26/26 paired subjects without exception. Total platelet count was equivalent. These changes reflect calcium chelation-induced cytoskeletal swelling, not genuine platelet hypertrophy, and are consistent with the functional impairment of growth factor release previously documented in vitro for EDTA-prepared PRP [11,12].

The clinical implications are direct and actionable. Platelet morphological parameters from EDTA samples—MPV, PDW, P-LCR, P-LCC, and PCT—are systematically overestimated by 7–26% relative to the physiological state, making EDTA-derived morphological data unreliable as a PRP quality indicator. Building on our group’s prior demonstration that patient-level biological variables independently modulate PRP composition [5,6], the present study adds the anticoagulant as a mandatory pre-analytical variable to account for when interpreting PRP quality data. For all PRP applications in regenerative and orthobiologic medicine, sodium citrate remains the anticoagulant of choice. Where EDTA is used pragmatically, morphological parameters must not be used to validate PRP quality, and rapid processing with CaCl_2_ activation is essential.

Future research should directly compare growth factor release capacity (PDGF-BB, TGF-β1, VEGF) and downstream clinical outcomes between EDTA- and citrate-prepared PRP to fully resolve the therapeutic implications of these morphological differences. Until that evidence is available, the present study provides a robust, clinically grounded, and effect-size-validated basis for anticoagulant selection in orthobiologic PRP therapy.

## Data Availability

Anonymised individual data are available from the corresponding author upon reasonable written request.
